# Preparation of G-CuO NPs and G-ZnO NPs with mallow leaves, investigation of their antibacterial behavior and synthesis of bis(indolyl)methane compounds under solvent-free microwave assisted dry milling conditions using G-CuO NPs as a catalyst

**DOI:** 10.3906/kim-2105-31

**Published:** 2021-10-19

**Authors:** Mine SULAK

**Affiliations:** 1 Department of Mathematics and Science Education, Faculty of Education, Pamukkale University, Denizli Turkey

**Keywords:** Indole, green synthesis, microwave reactions, antibacterial, zinc and copper oxide nanoparticles

## Abstract

In this study, biogenic copper and zinc oxide nanoparticles (G-ZnONPs and G-CuONPs) were synthesized by the green synthesis method using *Malva parviflora* L. (Millow) leaf extract and the obtained nanoparticles were characterized in detail with UV-Vis, FTIR, SEM, XRD. The antibacterial properties of the synthesized nanoparticles on gram-positive and gram-negative bacteria were investigated and it was found that the nanoparticles had high antimicrobial activity in the results of the experiments. With the obtained G-CuONPs, the synthesis of bis(indolyl)methanes with the “green” one-pot synthesis using microwave was achieved quickly and with high efficiency, and the thermal behavior of the obtained products was investigated.

## 1. Introduction

Nanotechnology is a science that deals with matter at a scale of 1 billionth of a meter (i.e. 10^–9^ m = 1 nm) and is also a field of study related to the manipulation of matter at the atomic and molecular scale [1]. Today, “Nano” is a popular term that is widely used in modern science and is also found in dictionaries: for example, nanoscience, nanowire, nanotube, nanotechnology, nanostructure, nanorobot, and nanomaterial. Nanoparticles have a wide range of use such as energy, physics, chemistry, biology, biotechnology, medicine, industry, technology, and industry [2,3]. Metallic nanoparticles (MNPs) are NPs with sizes between 1 and 100 nm [4]. Recently, impressive advances have emerged in the use of metal nanoparticles over biomolecular interactions, bioassays, biomedical devices, and a variety of other biomedical applications such as immunodiagnostics, drug delivery, therapeutics, and gene therapy. Zinc oxide (ZnO) nanoparticles, the most preferred among metal oxide nanoparticles, are available as white powders and dispersions. ZnO structures have many applications due to their biocompatibility, chemical photochemical stability, wide band gap of 3.37 eV and high binding energy (60 meV) as an important semiconductor [5]. 

The other nanoparticle used in the study is CuO. Available in different sizes and shapes among various metal oxide nanostructures, CuO is used in many application areas. Examples include sensors, cosmetics, antibacterial activity, chemical absorption, electrical and optical devices, catalysts for liquid phase hydrogenation, and catalyst applications for photocatalytic degradation, biomedical, textile, organic coatings [6–9]. CuO and ZnO nanoparticles are synthesized using chemical and physical methods [10]. Sol-gel process, chemical precipitation, hydrothermal method, microwave, and chemical vapor deposition are among these synthesis methods [11].

However, these methods involve the use of hazardous reagents for the synthesis of nanoparticles and often produce toxic materials that can be harmful to the environment [12, 13]. Therefore, it has become important to produce nanoparticles using low-cost, nontoxic, simple, fast, environmentally friendly methods (green chemistry). At this point, the green-synthesized CuO NPs (G-CuO NPs) and ZnO NPs (G-ZnO NPs) have recently gained increasing attention. The microorganisms used in the green synthesis method include algae, fungi, enzymes, herbs or plant extracts, polyphenols, and flavonoids with strong reducing potential and can act as both reducing and fixing agents in the synthesis process [14–16].

Plants called “medicinal plants” are frequently used in the production of nanoparticles with green synthesis method [17]. One of these medicinal herbs is the mallow plant belonging to the family Malvaceae. One of the mallow species is *M. parviflora* L., which grows mostly in the Aegean and Mediterranean regions. People in Turkey use mallow in the treatment of boils and wounds on the skin, externally as oatmeal porridge, and also for irritation and inflammation of the respiratory and digestive system. The reason why *M. parviflora* L. (Mallow) has these properties is a phytochemical compound called “Malvidine” [18]. In addition, the thymokine phytochemical compounds in the structure of *M. parviflora* L. (Mallow) are involved in the synthesis of nanoparticles.

Heterocyclic compounds are the building blocks of many biologically active substances, which have been studied by many organic chemists. Among such diverse heterocyclic compounds, bis(indolyl)methanes have a special place [19,20]. Indole skeletons can be found as an ingredient in many natural products with high structural complexities and biologically active molecules. As a result, indole and indole derivatives are constantly used in various research fields, e.g., pharmaceuticals, fragrances, agrochemicals, pigments, and materials science. Reserpine, ellipticalin, vincristine, cytotoxic euditalbin, dihydroflustramine, and dipyromethanes are some examples of indole derivatives. These compounds are used in antitumors, chemotherapy to cure cancer, antimicrobials, and antiparasites. These aforementioned indole derivates can be obtained through various methods, more specifically, dipyyromethan can be synthesized using several methods. Thus, in the literature, it was reported that there are many synthetic methods to prepare dipyyromethan and its derivates, such as reacting indole with different aldehydes in the existence of a catalysts like H_3_PMo_12_O_40 _[21], NaBArF_4 _[22], La(NO_3_)_3_ x6H_2_O [23], Fe(DS)_3 _[24] , HClO_4 _[25], ZrOCl_2_x8H_2_O [26], BiOClO_4_xH_2_O [27], ZrO_2 _[28, 29]. 

Conversely, disadvantages of these methods were reported in the literature as length of reaction time [30,31], high temperature [32,33],** use of expensive catalysts**, or harmful solvent. Use of microwave technology in organic chemistry has been extensively researched in the last two decades, and numerous publications have shown that many chemical syntheses can be successfully carried out with microwave [34,35].

Most importantly, the microwave technique shortens the reaction time considerably, as well as allowing high efficiency, less by-product formation, easier operation in harmony with green chemistry, solvent-free organic transformations, atomic economy, and selective reactions.

Although good results are obtained with both approaches in obtaining indole derivatives, these processes are time consuming. However, in microwave technique, the reaction takes place in a very short time [36].

In the present study, I synthesized CuO and ZnO nanoparticles using mallow plant leaves and examined their antibacterial activities. In addition, I am interested in the study of the synthesis of different substituted dipyyromethane (bis(indolyl) methane) by microwaving technique using CuO nanoparticles as catalysts.

## 2. Materials and methods

### 2.1. Materials

Copper sulphate hexahydrate (CuSO_4_ x6H_2_O 99.99 %), zinc acetate hepta hydrate (Zn (CH_3_COO)_2_ x6H_2_O 99.99 %), sodium hydroxide (NaOH), indole, and aldehyde derivatives were purchased from Germany (Sigma Aldrich Pvt. Ltd. ). As it is of analytical grade, all chemicals were used directly without further purification. The mallow plant (ebegümeci in Turkish) was collected from fields far from the industrial zone in Pamukkale District of Denizli Province. Bacteria used were obtained from Pamukkale University Biomedical Engineering Department.

### 2.2. Preparation of mallow leaf extracts

Mallow plant leaves were extracted according to the method suggested by Shu [37]. The leaf part of the mallow plant was removed and washed 3 times with deionized water. The leaves were passed through a food processor and divided into small pieces. Twenty grams of mallow leaves and 400 mL of deionized water were added into 1 L of erlenmayer. This mixture was heated in a magnetic heater at 100 °C for 2 h. The resulting mixture was filtered through Whatman (Grade GF / B: 1.0 µm) filter paper to obtain the extract. The extract was freshly prepared and used in the synthesis phase 

### 2.3. Synthesis of copper oxide nanoparticles

Copper oxide nanoparticles were synthesized by the phenolic compounds of the leaf extract through reduction of copper sulphate. By adding 2.6 g of CuSO_4_.5H_2_O onto 25 mL of mallow extract, the reaction was stirred continuously at 70 °C for 2 h. The pH was adjusted to 11 with 0.1 M NaOH, the reaction mixture changed from dark green to dark brown. The brown solid product was collected by centrifugation at room temperature and washed several times with distilled water. The product was dried overnight at 80 °C and then heated in an oven at 400 °C for 2 h [38].

### 2.4. Synthesis of zinc oxide nanoparticles

In a 100 mL beaker, 20 mL of Zn(CH_3_COO)_2_ x6H_2_O solution (10 mM) was added. Next, 5 mL of mallow extract was added dropwise onto this mixture. It was stirred at 80 °C until the color change. The mixture was taken into falcon tubes and centrifuged at 10000 rpm for 10 min. The resulting pellet was washed three times with water to remove organic residues and then centrifuged again. The pellet part was removed and dried in an oven at 80 °C for 12 h, it was then calcined in an oven at 400 °C. The nanoparticles obtained went through characterization processes and were stored in eppendorf tubes for use in antimicrobial studies [39].

### 2.5. Synthesis of bis(indolyl)methanes by conventional method

The dipyyromethanes (1a-k, Table 1) were prepared in 80%–91% yields by the condensation of the indole (2 mmol) with the appropriate aryl, alkyl aldehyde (1 mmol) in methanol in the presence of potassium hydrogensulphate (%5 mol). These compounds were isolated as solids and were stable both in solid state and in solution [40].

**Table 1:** Physical data of dipyyromethan derivatives and comparative study of conventional vs. microwave method.

Aldehyde	Compound	MP (°C)	Conventional method		MP (°C)	Microwave method	
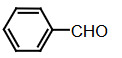	1a	142–144	Time (min.)	% yield	140-142	Time(min.)	% yield
			90	91		0.5	99
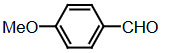	1b	193	120	83	194	1	96
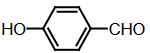	1c	200-210	180	89	210–215	1.5	95
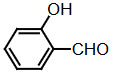	1d	258	150	88	260	1	97
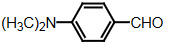	1e	203	120	85	200	2	93
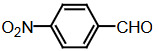	1f	240	210	89	242–245	3	92
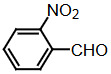	1g	212	180	76	222	2	90
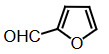	1h	145-147	120	85	142–144	1.5	94
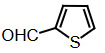	1i	125	150	86	127	1.5	99
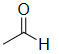	1j	100	150	89	98	1	96
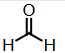	1k	85	120	90	88	1.5	98

### 2.6. Synthesis of bis(indolyl)methanes with the microwave method

Benzaldehyde (1 mmol) and indole (2 mmol) were mixed, and G-CuONPs (20 mg) was added. The mixture was ground with a mortar and a pestle until all reactants were completely mixed with each other. The mixture was poured into a beaker, which was then placed in a microwave (LG, MH-4048GW, 480 W, 2 min)

After the reaction was completed, the mixture was allowed to cool to room temperature. We then added approximately 5 mL of ethyl acetate. The CuO nanoparticle was removed by centrifuge, and the ethyl acetate evaporated. Precipitate of the desired compound was recrystallized from ethanol.

**Compound 1a** = Solid; mp 194–196 °C; FTIR (KBr), cm^–1^, nmax: 1031 (C-N), 1460 (Ar-C=C), 2930 (Alif-CH), 3055 (Ar-CH), and 3393 (NH); ^1^H NMR (500 MHz, DMSO- *d6*): 5.75 (s, 1H, Ar-CH), 6.80 (s,2H, Ar-H), 7.00 (t, 2H, Ar-H), 7.15–7.28 (m, 10H, J¼8.1 Hz, Ar-H), 10.9 (s, 2H, NH).

**Compound 1b** = Solid; mp 194 °C; FTIR (KBr), cm^–1^, nmax: 1250 (C-O-C), 1420 (C-N), 1510–1620 (Ar-C=C), 2890 (Ar-OCH_3_), 3060 (Ar-CH), and 3390 (NH); ^1^H NMR (500 MHz, DMSO- *d6*): 3.68 (s, 3H, O-CH_3_), 5.85 (s, 1H, Ar-CH), 6.64 (s, 6H, Ar-H), 6,83(d, 2H, Ar-H) 7.00 (t, 2H, Ar-H), 7.12 (t, 2H, Ar-H), 7.30–7.38 (dd, 2H, J¼8.1 Hz, Ar-H), 10.86 (s, 2H, NH).

**Compound 1c** = Solid; mp 210–215 °C; FTIR (KBr), cm^–1^, nmax: 1220 (C-O), 1459 (C-N), 1500–1616 (Ar-C=C), 3054 (Ar-CH) and 3410 (NH), 3600 (OH); ^1^H NMR (500 MHz, DMSO-*d6*): 5.72 (s, 1H, Ar-CH), 6.58 (s, 2H, 8.5 Hz, Ar-H), 6.83 (t, 2H, J¼1.8 Hz, ArH), 7.06 (t, 2H, Ar-H), 7.20–7.34 (m, 8H, J¼7.9 Hz, Ar-H), 9.21 (s, 2H, NH), 10.5 (s, 1H, OH).

**Compound 1d** = Solid; mp 260 °C; FTIR (KBr), cm^–1^, nmax: 1240 (C-O), 1455 (C-N), 1490–1699 (Ar-C=C), 3050 (Ar-CH) and 3420 (NH); ^1^H NMR (500 MHz, DMSO-*d6*): 6.20 (s, 1H, Ar-CH), 6.78–7.12 (m, 9H, Ar-H), 7.32 (dd, 4H, J¼7.8 Hz, Ar-H), 7.38 (dd, 1H, J¼8.2 Hz, Ar-H), 9.48 (s, 2H, NH), 10.87 (s, 1H, OH).

**Compound 1e =** Solid; mp 200 °C; FTIR (KBr), cm^–1^, nmax: 1190 (Ar-N), 1465 (C-N), 1599 (Ar-C=C), 3070 (Ar-CH) and 3430 (NH); ^1^H NMR (500 MHz, DMSO-*d6*): 2.91 (s, 6H, CH_3_), 5.71 (s, 1H, Ar-CH), 6.71 (d, 2H, J¼8.7 Hz, Ar-H), 6.83 (d, 2H, J¼2.0 Hz, Ar-H), 6.91 (s, 2H, Ar-H), 7.11 (s, 2H, Ar-H), 7.21 (d, 2H, J¼8.6 Hz, Ar-H), 7.31 (d, 2H, J¼7.9 Hz, Ar-H), 7.37 (d, 2H, J¼8.1 Hz, Ar-H), 10.89 (s, 2H, NH).

**Compound 1f =** Solid; mp 242–244 °C; FTIR (KBr), cm^–1^, nmax: 1259 (C-N), 1352 (N-O), 1540 (Ar-N), 1598–1616 (Ar-C=C), 3049 (Ar-CH) and 3451 (NH); ^1^H NMR (500 MHz, DMSO-*d6*): 6.00 (s, 1H, Ar-CH), 6.91 (s, 2H, Ar-H), 7.09–7.15 (m, 3H, Ar-H), 7.32 (d, 3H, J¼8.0Hz, Ar-H), 7.40 (d, 2H, J¼8.1Hz, Ar-H), 7.73 (d, 2H, J¼8.7Hz), 8.22 (d, 2H, J¼8.7Hz Ar-H), 10.9 (s, 2H, NH).

**Compound 1g =** Solid; mp 222 °C; FTIR (KBr), cm^–1^, nmax: 1245 (C-N), 1348 (N-O), 1481–1614 (Ar-C=C), 3065 (Ar-CH) and 3448 (NH); ^1^H NMR (500 MHz, DMSO-*d6*): 6.4 (s, 1H, Ar-CH), 6.67 (dd, 2H, Ar-H), 7.04 (dt, 2H, Ar-H), 7.24 (dt, 2H, J¼7.4Hz, Ar-H), 7.3 (d, 2H, J¼8.12Hz, Ar-H), 7.49 (d, 2H, J¼7.12Hz, Ar-H), 7.59 (t, 1H, Ar-H), 7.61 (d, 1H, J¼7.8Hz, Ar-H), 8.01 (dq, 1H, J¼8.7Hz, Ar-H), 8.23 (dq, 1H, J¼8.7Hz, Ar-H), 10.5 (s, 2H, NH).

**Compound 1h =** Solid; mp 142–144 °C; FTIR (KBr), cm^–1^, nmax: 1461–1620 (Ar-C=C), 1709 (C-O), 3058 (Ar-CH) and 3421 (N-H); ^1^H NMR (500 MHz, DMSO-*d6*): 5.89 (s, 1H, Ar-CH), 6.17–7.10 (m, 5H, Ar-H) 7.21–7.31 (m, 6H, Ar-H), 7.41 (d, 2H, J¼8.1 Hz, Ar-H), 9.8 (s, 2H, NH).

**Compound 1i =** Solid; mp 127 °C; FTIR (KBr), cm^–1^, nmax: 600 (Ar-S), 1451–1622 (Ar-C=C), 1719 (C-S), 3034 (Ar-CH) and 3420 (N-H); ^1^H NMR (500 MHz, DMSO-*d6*): 6.1 (s, 1H, Ar-CH), 6.83-6.95 (m, 4H, Ar-H), 7.0–7.13 (m, 4H, Ar-H), 7.30 (dd, 1H, J¼3.6 Hz, Ar-H), 7.37 (dd, 4H, J¼ 11.4Hz, Ar-H), 8.5 (s, 2H, NH).

**Compound 1j =** Solid; mp 98 °C; FTIR (KBr), cm^–1^, nmax: 1381 (CH_2_), 1456–1630 (Ar-C=C), 2910 (CH_3_), 3050 (Ar-CH) and 3431 (N-H)_;_^1^H NMR (500 MHz, DMSO-*d6*): 1,6 (d, 3H, CH_3_-CH), 4,5 (q, 1H, J¼7.3 Hz, CH-CH_3_), 7.0 (m, 2H, J¼ 6.9 Hz, Ar-H), 7.2 (m, 2H, J¼8.1 Hz, Ar-H), 7.41 (dd, 2H, Ar-H), 7.67 (d, 2H, Ar-H), 8.3 (s, 2H, NH).

**Compound 1k =** Solid; mp 88 °C; FTIR (KBr), cm^–1^, nmax: 1430 (CH_2_), 1610 (Ar-C=C), 3100 (Ar-CH) and 3490 (N-H); ^1^H NMR (500 MHz, DMSO-*d6*): 4.20 (s, 2H, CH_2_), 6.88 (m, 2H, Ar-H), 7.00 (m, 2H, Ar-H), 7.20 (d, 2H, J¼2.0 Hz, Ar-H), 7.40 (d, 2H, J¼8.0 H, Ar-H), 7.65 (d, 2H, J¼7.9 Hz, Ar-H), 8.1 (s, 2H, NH).

### 2.7. Characterization methods of compounds

#### 2.7.1. Characterization methods of nanoparticles 

The synthesized NPs were enclosed in a quartz cuvette and the absorbance was measured in the wavelength range of 200 to 800 nm using a UV-Vis spectrophotometer (UV / Vis-1601, Shimadzu, Kanagawa, Japan) adjusted to a resolution of 1 nm.

Fourier transform infrared spectroscopy (FTIR) shows the presence of bio-organic components that act as a possible stabilizer for synthesized NPs. After the synthesis of the NPs, biomolecules associated with it were identified by FTIR measurements.

 G-CuONPs and G-ZnONPs were subjected to FTIR analysis. The samples were exposed to an infrared source with the spectrum scanned in the 400–4000 cm^–1^ range to achieve good signal to noise ratio. Various vibration modes have been identified and assigned to identify different functional groups on the NPs.

Chemical composition analysis and morphology of the synthesized NPs were performed using an energy-dispersive X-ray spectroscopy (EDX) analyzer associated with a scanning electron microscope (SEM) (Zeiss Supra 40 VP). A very small sample of the synthesized and stabilized NPs was prepared on a carbon-coated copper grid, and then SEM imaging was taken by coating with a layer of gold-palladium using the spray coater (Quorum Q150R ES, Quorum Technologies Ltd, UK).

The nanoparticle powder obtained was analyzed using an ADP PRO 2000 X-ray diffraction system with Cu Kα radiation (λ =1.54059 Å). The XRD pattern was studied with a 0.02 step size in the 2θ range between 5**° and **90°. The area size of the crystal was calculated using the Debye–Scherrer formula [41].

#### 2.7.2. Characterization methods of bis(indolyl)methane derivatives

Melting points were measured on an electrothermal digital microscopic melting point apparatus and recorded without modification. The reaction was followed by thin layer chromatography on silica. ^1^H NMR and ^13^C NMR spectra were recorded with tetramethylsilane as standard in CDCl_3_ or DMSO-*d6* solution, respectively, using a Bruker AMX 500-MHz spectrometer at 500 and 125 MHz. Chemical shifts were measured in parts per million (ppm) and coupling constants (J), hertz (Hz).

Low resolution mass spectrographic analyses were measured using the electrospray ionization technique on a Bruke Esquire 3000 spectrometer. Thermogravimetry (TG), differential thermogravimetry (DTG), and differential thermal analysis (DTA) curves were obtained simultaneously using a Shimadzu DTG-60H thermal analyzer. The air atmosphere was selected and the flowing rate was adjusted as 25 mL min^–1^, since the potential applications of these compounds were carried out in air. All compounds were heated from room temperature to 800 °C with using 10 °C min^–1^ heating rate. Silver was used to calibrate the balance for buoyancy effects for the quantitative estimation of mass change. The melting points indium of tin (provided by Shimadzu) were used to calibrate the temperature. Four different compounds (1a**, **1b**,** 1d**,** 1h) were selected for thermal investigation. These compounds were chosen due to the varied functional groups. 

### 2.8. Antimicrobial activity

In this study, the antimicrobial activity of nanoparticles was defined on three gram- negative and five gram-positive bacteria using disk diffusion method according to Clinical Laboratory Institute standard Anonymous (2020). M100 | Performance Standards for Antimicrobial Susceptibility Testing, 31st Edition, Clinical Laboratory Institute [online]. Website https://clsi.org/

Paper discs impregnated with certain amounts of G-CuONps and ZnO NPs were placed in solid media with a high concentration of the microorganism to be tested using a pair of sterile forceps. During this process, it has been paid attention that there is a distance of 22 mm between the discs and 14 mm from the edge of the petri dish so that the zones to be formed do not overlap each other. Media were incubated for 18–24 h at 35 °C. The diameter of the inhibition zone was measured in millimeters and evaluations were made according to standard tables.

## 3. Results and discussion

Dipyyromethane (bis(indolyl)methane) was synthesized using both conventional methods and microwaves, and the same material was used in both conditions. 

**Figure 1:**
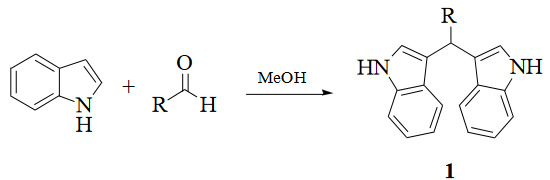
Conventional methods of synthetic reactions.

**Figure 2:**
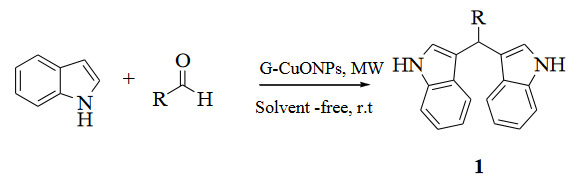
Microwave methods of synthetic reactions.

Traditional synthesis methods in organic chemistry require a longer heating period and therefore also require an exhaustive and tiresome apparatus set-up which results in a higher cost and environmental pollution (Figure 1). Additionally, the synthesis process requires large quantities of toxic chemicals and solvents for reactions. Reactions with microwave are a study of “green chemistry” performed in closed containers at high temperatures with low-boiling solvents (Figure 2). Green processes use less catalysts and readily recyclable solvents or media and often yield more products than normal. When the reactions with microwave and traditional organic chemistry were compared, it was observed that the product yield increased by 10%–30% compared to traditional methods. Each reaction was repeated at least three times (with different time intervals). Comparative results regarding the products’ melting points and percentage yields are presented in Table 1. The reaction with indoles has been demonstrated using various substituted aromatic and aliphatic aldehydes. Bis(indolyl)methanes were obtained in 90% and higher yields. The substituents in the aromatic ring, whether electron withdrawing or electron donating, (these substituent groups were in the o-, m-, p- positions on the ring) did not show any visible effect on the formation of the bis(indolyl)methane compound, and the products were obtained with high yield.

**Figure 3:**
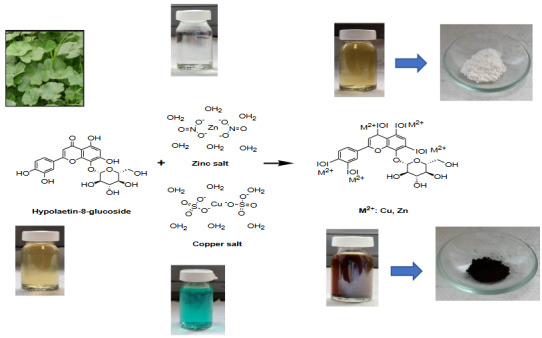
Bioextract of the mallow plant, Zn, Cu salt solution, G-CuONPs and G-ZnONPs solution.

Especially in medicinal plants, there are many metabolites with pharmacological activity. Many studies have shown that these metabolites become more effective by binding to the synthesized nanoparticles and add more properties to nanoparticles [42]. Another advantage of the green synthesis of nanoparticles is that while some functional groups must be added to the surface of nanoparticles in physicochemical synthesis, this step is not necessary for nanoparticles synthesized in biological ways [43]. In addition, the time required for the biosynthesis of nanoparticles is shorter than the time required for physiochemical approaches. As a result of the interaction of plant extract and metal (Cu, Zn) salts, the color changes occurring in the reaction medium and metal oxide nanoparticles were obtained (Figure 3).

**Figure 4:**
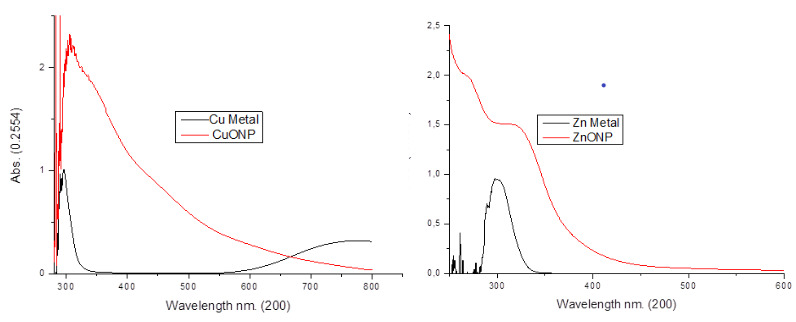
Uv-Vis spectrum of ZnO and CuO nanoparticles.

UV-Visible spectroscopy was used to examine the optical properties of CuO and ZnO nanoparticles obtained as a result of experimental studies. In Figures 4a and 4b, UV-Visible spectra of CuO and ZnO nanoparticles prepared by green synthesis are given. Transition type and band gap value can be determined from absorption bands in UV-Visible spectroscopy [44,45].

A characteristic absorption peak at 360 nm wavelength is given in the UV-Visible spectrum of G-ZnO nanoparticles in Figure 4b. This base band absorption of ZnO is due to electron transitions from the valence band to the conducting band.

The band gap energy (E) of the prepared CuO and ZnO nanoparticles was calculated using the following equation (Equation 1) [46].

E = h × C/λ (1)

Here, E = Band gap energy

h = Planck’s constant = 6.626 × 10^–34^ Joule sec

C = Speed of light = 3.0 × 10^8^ meters / sλ = Cut wavelength = 299× 10^–9^ meters (ZnO)294× 10^–9^ meters (CuO)

* Conversion 1eV = 1.6 × 10^–19^ Joules

The band gap energy (E) of the prepared ZnO and CuO nanoparticles were calculated using equation (1). The calculated band gap energy value of ZnO nanoparticles is 4.16 eV. It reveals that ZnO nanoparticles can be used in medical applications such as sunscreens or antiseptic ointments due to their intense absorption in the UV region. The band gap energy of the CuO nanoparticle obtained by the green synthesis method from the mallow plant is 4.22 eV. Since the G-CuO NPs energy band gap is more than 4 eV, it can be used as a photocatalyst.

FTIR analysis performed to characterize the surface structure of G-CuNPs is shown in Figure 5a. The visible bands belong to the functional groups of biomolecules adsorbed on NPs. FTIR spectra of G-CuNPs exhibited vibrations in the region of 500–600 cm^–1^ that can be attributed to Cu vibrations confirming the formation of G-CuNPs. Depending on the vibrations of Cu, an absorption band was observed at 625 cm^–1^. A dense and wide band of 3303 cm^–1^ emerged in the region corresponding to the stretching movements of hydroxyl functional groups [47]. Metal-oxygen (Cu-O) band vibrations were determined at 515 cm^–1^.

**Figure 5a:**
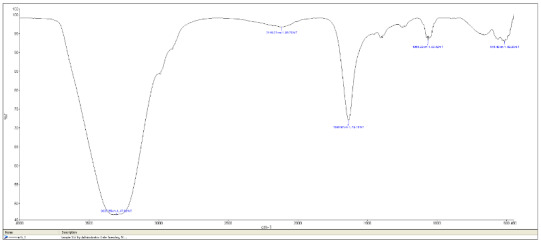
FTIR analysis of CuO nanoparticles.

**Figure 5b:**
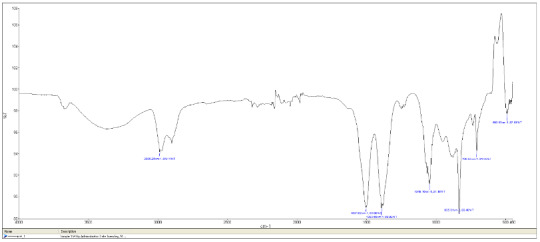
FTIR analysis of ZnO nanoparticles.

In the FTIR spectra of the synthesized ZnO nanoparticle, a strong band was observed at 470 cm^–1^. It has been reported in the literature that this absorption band belongs to the characteristic vibration of the Zn-O bond (Figure 5b). When the FTIR spectra of G- ZnO NPs given in the literature were examined, it was determined that the FTIR spectra obtained were compatible with the spectra given in the literature [48].

SEM images of synthesized ZnO nanoparticles are given in Figure 6a. When SEM images are examined, it is seen that the produced ZnO particles generally have a hexagonal morphological structure. ZnO nanoparticles obtained by the green synthesis method have an average diameter of 64.5 nm.

**Figure 6a:**
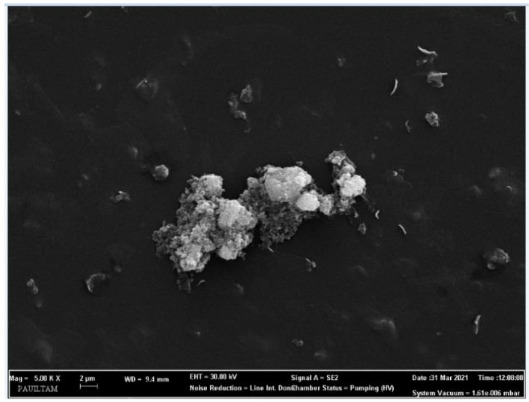
SEM image of ZnO nanoparticles.

**Figure 6b:**
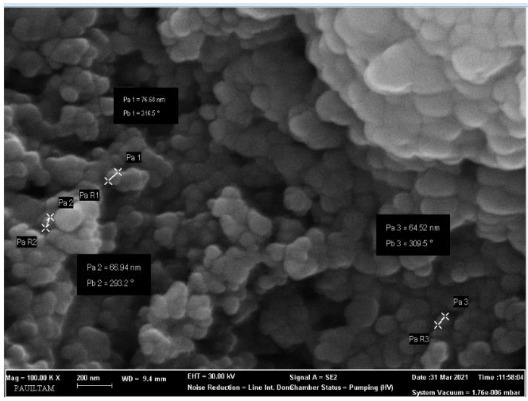
SEM image of CuO nanoparticles.

When the SEM images were examined, it was observed that the synthesized G-CuONPs had spherical shapes. It was observed that the particle sizes for the synthesized G-CuONPs were in the range of 42.2–47.2 nm (Figure 6b).

EDX analysis was performed to examine the elemental composition of metal oxide nanoparticles obtained as a result of experimental studies. EDX analysis of CuO and ZnO nanoparticles obtained are given in Figures 7a and 7b.

**Figure 7a:**
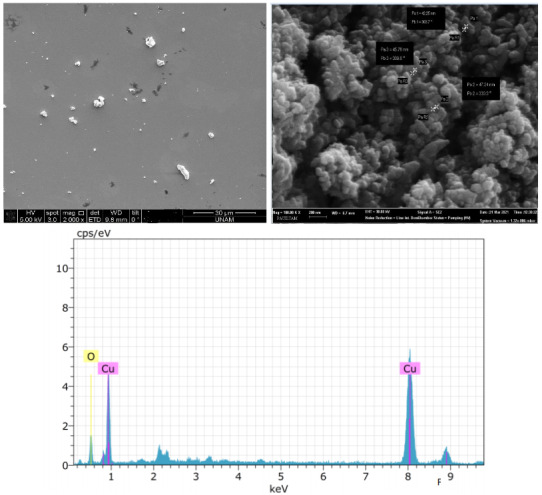
EDX spectrum of CuONp synthesized with bioextract.

**Figure 7b:**
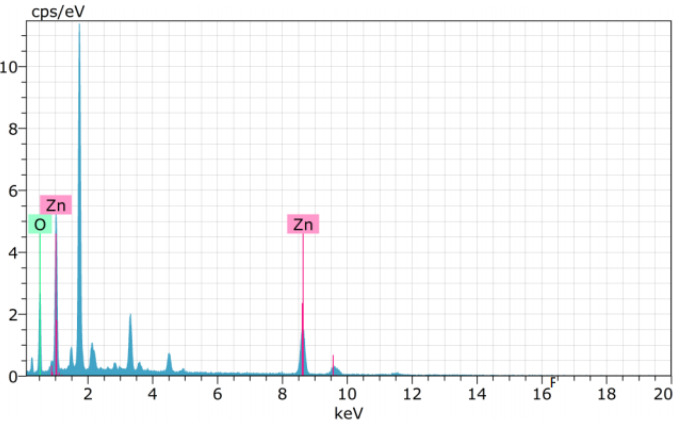
EDX spectrum of ZnONp.

Theoretically, the atomic percentage of metals (Cu and Zn) and oxygen should be 50% each. As seen in Figure 7a, as a result of the analysis, it was determined that the atomic copper percentage was 50% and the oxygen percentage was 50%. As can be seen in Figure 7b, as a result of the analysis, it was determined that the atomic percentage of zinc and oxygen were 50% and 50%, respectively. When looking at EDX analysis of both metal oxide nanoparticles, it is seen that they are stoichiometric and compatible with theoretical values.

According to the results of XRD analysis, it was determined according to the ICDD library that the peaks belong to the monoclinic CuO nanoparticles, and the mesh parameters were a = 4.687 Å b = 3.422 Å c = 5.130 Å (ICDD card no: 01-089-5897). The average particle size calculated according to the Debye–Scherrer formula is approximately 25 nm. In Figure 8a, the X-ray diffractogram of the G-CuONPs sample is given with Miller indices (orientation planes).

**Figure 8a:**
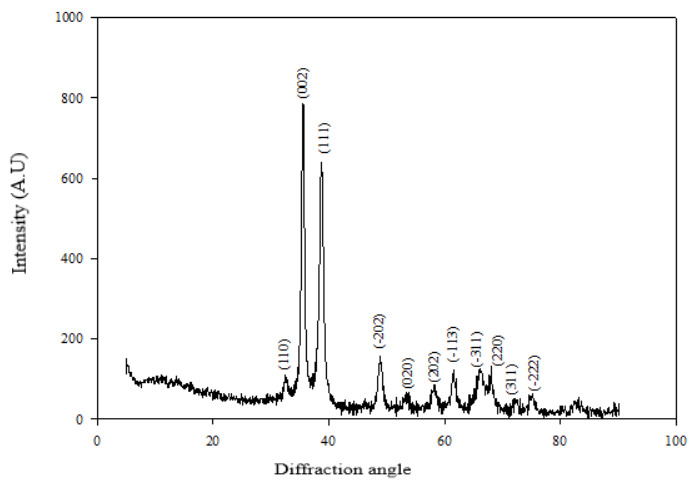
X-Ray Diffractogram / Diffraction Pattern of nano CuO compound derived from malow plant

**Figure 8b:**
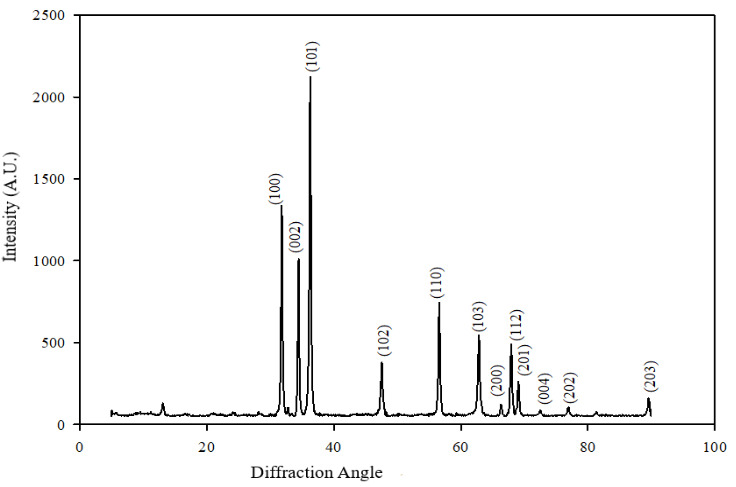
X-Ray Diffractogram / Diffraction Pattern of nano ZnO compound derived from malow plant

ZnO structural characterization using X-ray diffractometry has been made. Figure 8b shows the XRD diffraction pattern of ZnO nanoparticles prepared by biosynthesis method. Diffraction peaks at a=3,250 Å ve c=5,207 Å 2θ correspond to the (100), (002) and (101) planes, respectively, confirming that ZnO has a hexogonal structure. There are no peaks attributable to any impurities and the expansion of the peaks, and this confirms that the synthesized ZnO nanoparticles are highly pure nanocrystal. The average particle size calculated according to the Debye–Scherrer formula is approximately 31.38 nm. 

Four different compounds (1a**,** 1b**,** 1d**,** 1h) were selected to examine the thermal decomposition stages and to compare thermal stability in this part of the study. It was found that thermal stabilities of compounds vary depending on the substituted groups. Compound 1b shows the highest thermal stability and starts to decompose after 175 °C. However, compound 1a shows the lowest thermal stability and starts to decompose after 130 °C. The thermo-analytical results are summarized in Table 2.

**Table 2:** The thermoanalytical results of all compounds (1a, 1b, 1d, 1h).

Compound	Ti–Tf/oC		Tpeak/oC	Mass loss%(exp.)	Mass loss%(theo.)
1a	130–305		287.77	23.358	23.602
	305–587		523.99	37.116	36.602
	587–802		628.44	43.371	39.796
1b	175–321		307.89	29.549	30.114
	321–527	419.58	31.953	32.954	
	527667	541.71	36.932	40.664	
1d	161–462	230.42	27.680	27.219	
	462–597597–654	535.05613.53	35.07234.319	34.31939.552	
1h	147–479	318.09	37.738	37.179	
	479–630	507.23	62.821	67.417	

**Figure 9:**
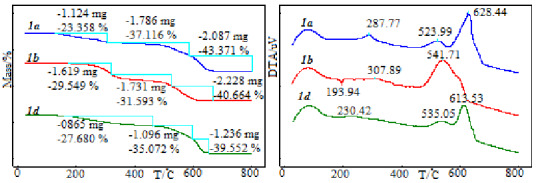
The TG and DTA curves of 1a, 1b, 1d compounds.

The first three compounds (1a**,** 1b**,** 1d) show similar thermal behavior. TG and DTA curves of these compounds are given in Figure 9. These compounds decompose with three exothermic stages. The first stages correspond to the removal of aldehyde groups. Experimental mass loss values (23.358, 29.549, and 27.680, respectively) of these stages are compatible with theoretical mass loss values (23.602, 30.114, 34.319). One indole group degrades from the structure at the second stage. The average reaction interval of this degradation is 363–570 °C. Finally, the rest of the structure is decomposed. Peak temperature of this stage is observed as 628.44, 541.71, and 613.53 °C, respectively. Differently from compounds 1a and 1d, an endothermic peak was observed in the DTA curve of compound 1b. This peak belongs to melting and shows a maximum at 193.94 °C. 

**Figure 10:**
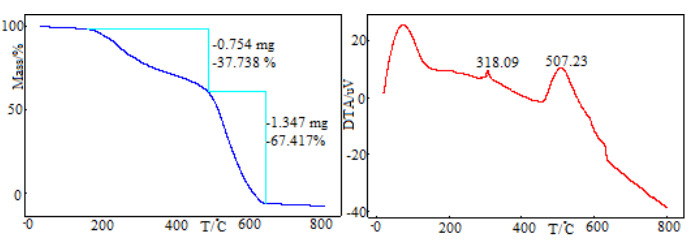
The TG and DTA curves of 1h compound.

The TG and DTA curves of 1h compounds are presented in Figure 10. The decomposition mechanism of this compound shows differences compared to the other three compounds. Decomposition proceeds with two exothermic stages. The first stage corresponds to the degradation of one indole group and occurs in the 147–479 °C temperature range, with 37.738% experimental mass loss. The second stage starts immediately after the completion of first stage and the decomposition of all structures continues. Exothermic characters of this stage are higher than the first reaction stage. The reaction peak temperature is 507.23 °C. 

Thermal stabilities and decomposition mechanisms of four bis(indolyl)methanes compounds were compared. As a result of these comparisons, we concluded that the thermal stability of the compounds depended on substituted groups and their positions. Determination of thermal stability of these compounds is very important for potential usage areas.

This experiment was carried out using solvent-free reaction conditions, various benzaldehyde derivatives, and indoles that reacted with CuO nanoparticle which is inexpensive and stable. In addition to the aromatic aldehyde, aliphatic aldehyde was used and obtained excellent results were obtained in aliphatic aldehydes. After completion of the reaction, the reaction mixture ethyl acetate (5 mL) and G-CuONPs were recovered by centrifuge, washing with methanol, and drying in a vacuum used in other reactions without losing effectiveness. I examined the synthesis of bis(indolyl)methanes performed both with the conventional method and the microwave method. I have found that, with the microwave method, products are obtained with higher efficiency and higher purity, greater reproducibility, lower energy use in a convenient, cleaner, and efficient way, and the synthetic protocol is efficient for using G-CuONPs under solvent-free conditions.

## 4. Antibacterial studies

Research has shown that bio-synthesized nanoparticles have higher antimicrobial activity compared to chemically synthesized nanoparticles. Mukherjee et al. reported that biological nanoparticles showed 96.67% antibacterial activity, but chemically synthesized nanoparticles did not show a significant effect on antibacterial activity [48].

As mentioned earlier, antibacterial properties of CuO and ZnO nanoparticles, three gram-positive bacteria (*B. subtilis*,* S. aureus*,* B. cereus*) and five gram-negative (*K. pneumoniae*,* V. parahaemolyticus*,* E. coli*,* S. typhimurium*,* S. entritidis*) were investigated using disk diffusion method against six bacterial strains. The antibacterial effects of ZnO and CuO nanoparticles against bacteria are given in Table 3.

**Table 3:** Inhibition zones formed by G-CuONPs and G-ZnONPs.

	K. pneumoniae	V. parahaemolyticus	E. coli	S. typhimurium	S. enteritidis	B. subtilis	S. aureus	B. cereus
CuO	3	13	14	2	4	11	2	8
ZnO	18	21	10	13	19	19	13	13

**Figure 11:**
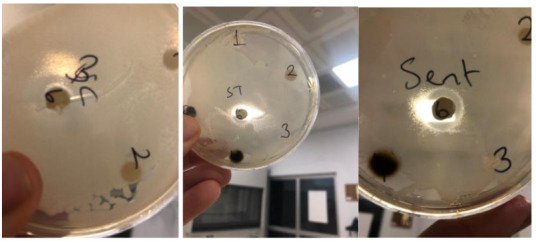
Antibacterial effects of G-CuONPs and G-ZnONPs against *B. subtilis, S. Aureus, B. Cereus, K. pneumoniae, V. parahaemolyticus, E. coli, S. typhimurium, S. entritis.*

In this study, in vitro susceptibilities of CuO and ZnO nanoparticles synthesized by green synthesis method against gram-positive and gram-negative bacteria were determined using disk diffusion methods. For two different nanoparticles, it was observed that it has different microbial activity against different bacteria at varying rates between 2 mm and 21 mm. In the disk diffusion test, the largest zone diameter for bacteria was measured against *V. parahaemolyticus* with a diameter of 21 mm using the ZnO nanoparticle (Figure 11). *Vibrio parahaemolyticus* is observed with a maximum zone diameter while the zone diameter is small against CuO nanoparticles *Klebsiella pneumoniae*, *Salmonella typhimurium*, and *Salmonella enteritidis*. Differences in sensitivity and resistance to both gram-positive and gram-negative bacterial populations may be due to differences in cell structure, physiology, metabolism, or the degree of contact of organisms with nanoparticles. In addition, other factors such as nanoparticle diffusion rate may affect the bacterial strain differently [49].

## 5. Conclusions 

Many researchers have published studies on the green synthesis of copper and zinc nanoparticles [50,51], but there is no literature on the green synthesis of G-CuO and G-ZnO NPs using the leaves of mallow plants grown in Denizli, indigenous to the Aegean region.

In the present study, I have successfully synthesized CuO and ZnO nanoparticles using mallow plant leaves and observed that CuO nanoparticles can be used as a catalyst. Especially in the synthesis of indole compounds with many applications in the field of medicine, I have obtained high yields in a very short time with the microwave method in a solvent-free environment by using CuO nanoparticles as catalysts. Nanoparticles and bis(indolyl)methanes have been obtained using a cost-effective, economical, environmentally friendly synthesis method that requires very short time.

Finally, both CuO and ZnO nanoparticles prepared by green synthesis have significant antibacterial effects and have a lethal effect on some bacteria tested in this study. The results show that CuO and ZnO nanoparticles can possibly be designed as an antibacterial agent that can be used in food protection, pharmaceutical industry, agriculture, and daily use [52,53]. 

## Funding

This research did not receive any specific funding.
